# The Restoration of Function after Section of Nerve Trunks

**Published:** 1900-02-24

**Authors:** 


### THE RESTORATION OF FUNCTION AFTER SECTION OF NERVE TRUNKS.

Nothing is more mysterious than the manner in which, after the section of a nerve trunk, function is in some cases quickly restored in the peripheral portion of the nerve. We are taught by physiological investigation that the individual nerve fibres are paths between well-defined centres and peripheral nerve endings, and it is clear that if this is true the restoration of function in a divided nerve, the cut ends of which are brought together in a more or less haphazard manner, is a most remarkable performance. It is out of the question to believe that it can ever happen, unless as the result of an extraordinary coincidence which cannot be expected to be repeated, that the corresponding ends of the cut fibrils can be brought into apposition; in fact, it is almost certain that in coapting the cut ends of the nerve most of the cut fibres must be brought in contact with ends which do not correspond with them, and thus it would be expected that in the reunited trunk nervous messages would go astray along all sorts of new and unaccustomed paths. But this is not what happens. Despite its complexity of structure and of function, the reunited nerve seems as capable as before division of subserving its functions. To ascertain if possible what is the process by which this restoration of function is effected Dr. Robert Kennedy[1] performed three experiments on the sciatic nerves of dogs. In two, after the nerve was divided, the peripheral segment before being united by sutures was partially rotated so as to bring a majority of non-corresponding fibres into juxtaposition, while in the third experiment the cut nerve was reunited as accurately in the old position as possible. In all the cases recovery of function followed a practically identical course, physiological examination showing that reunion of the nerve had taken place both anatomically and physiologically. On examining the cut nerves it was found that while the central segment except for some dilatation of the lymph spaces, showed the character of normal sciatic nerve, the peripheral segments showed no old nerve-fibres in any part examined, either close to the seat of union or in the terminal divisions of the nerves. In their place were abundant young nerve-fibres with well-defined characters, and lying between the young nerve-fibres were degenerated remains of the old fibres, showing that Wallerian degeneration had taken place. In no case could a nerve be traced in any one section from one side to the other of the cicatrix, and the arrangement of the young fibres in the cicatricial sections was very tortuous and irregular, resembling much what is seen in a neuroma. Thus it appears that, after section and immediate coaptation of a nerve, restoration of conductivity and of voluntary function may be effected in a few days; that this early restoration need not be the result of reunion of old nerve-fibres, but may be the result of regeneration of young nerve-fibres in the peripheral segment; that voluntary co-ordinated movements are regained equally soon whether the two ends of the divided nerves are united as accurately as possible, so as to bring the corresponding ends of the nerve-fibres into contact as nearly as possible, or whether previous to reunion the peripheral segment is twisted so that non-corresponding nerve-fibres come in contact; and thus that in suturing a divided nerve no trouble need be taken to secure that the coaptation of the ends takes place in the old position—a very useful and practical bit of teaching.

[1] Transactions of the Royal Society of Edinburgh, 1899.

